# Alteration of non-protein respiratory quotient after hepatocellular carcinoma treatment can be related to des-γ-carboxy prothrombin before treatment

**DOI:** 10.1186/2193-1801-1-55

**Published:** 2012-11-29

**Authors:** Masaya Saito, Yasushi Seo, Yoshihiko Yano, Akira Miki, Kenji Momose, Hirotaka Hirano, Masaru Yoshida, Takeshi Azuma

**Affiliations:** 1 Division of Gastroenterology, Department of Internal Medicine, Kobe University Graduate School of Medicine, 7-5-1, Kusunoki-cho, Chuo-ku, Kobe, 650-0017 Japan; 2 Center for Infectious Diseases, Kobe University Graduate School of Medicine, Kobe, Japan; 3 Division of Metabolomics Research, Department of Internal Medicine, Kobe University Graduate School of Medicine, Kobe, Japan

**Keywords:** Des-γ-carboxy prothrombin, Energy malnutrition, Hepatocellular carcinoma, Hypoxic stress, Indirect calorimetry, Non-protein respiratory quotient, Transcatheter arterial chemoembolization

## Abstract

**Background:**

Transcatheter arterial chemoembolization (TACE) is an effective treatment for hepatocellular carcinoma (HCC) that would occasionally lead to energy malnutrition through therapeutic hypoxic stress. We aimed to clarify the correlation between the energy malnutrition after TACE and low tolerability for hypoxia of non-tumoral liver before TACE.

**Findings:**

We performed a prospective cohort study involving 100 HCC patients who underwent TACE at Kobe University Hospital. Indirect calorimetry was performed before and 7 days after TACE, and non-protein respiratory quotient (npRQ) as an indicator of the energy malnutrition was measured. Blood biochemical examinations were also performed before TACE. As an indicator of hypoxic marker, des-γ-carboxy prothrombin (DCP) was measured before TACE. The correlation between npRQ ratio (7 days after/before TACE) and DCP (before TACE) was statistically examined. Spearman’s correlation coefficient test showed that npRQ ratio (Day 7/Day 0) was significantly related to DCP (Day 0) (p=0.0481, r=-0.2033). On the other hand, npRQ ratio (Day 7/Day 0) was not related to alpha fetoprotein (Day 0) (p=0.6254, r=-0.0494).

**Conclusions:**

The npRQ reduction after TACE was related to a high value of DCP before TACE. The energy malnutrition after TACE would originate from low tolerability for hypoxia of non-tumoral liver. The HCC patients with a high value of DCP before TACE would clinically have a high risk of the energy malnutrition after TACE.

## Findings

Transcatheter arterial chemoembolization (TACE) is an effective treatment for hepatocellular carcinoma (HCC) that can deteriorate liver function. The long-term deterioration of liver function after TACE can originate from the progression of energy malnutrition (Saito et al. 2012a). In general, the non-protein respiratory quotient (npRQ) is the main factor used to evaluate energy metabolism on the basis of indirect calorimetry (Tajika et al. 2002). Short-term reduction in npRQ was found to be related to long-term liver dysfunction after TACE (Saito et al. 2012a). On the other hand, no background characteristic factors that could promote the development of energy malnutrition after TACE have been identified. We aimed to clarify the background factors related to npRQ reduction after TACE.

We performed a prospective cohort study on a total of 100 patients (mean age: 70.9 years, range: 41-87 years; male:female ratio: 61:39) who underwent TACE for HCC. All patients were Japanese and had liver cirrhosis. Overall, 58 patients were classified into Child’s grade A and 42 were classified into grade B. The etiology of cirrhosis was as follows: hepatitis B in 12 patients, hepatitis C in 71, alcoholic liver dysfunction in 28, primary biliary cirrhosis in 2, autoimmune hepatitis in 2, non-alcoholic fatty liver disease in 5, and unknown in 1, although each category overlaps with others. Blood biochemical examinations were performed before TACE and indirect calorimetry was performed before and 7 days after TACE. The therapeutic volume was calculated from the distribution of Lipiodol deposits on a liver CT scan after TACE. The relationship between two variables was investigated by Spearman’s correlation coefficient test. Logarithmic transformation was performed only when variables displayed skewed distributions.

**Figure 1:**
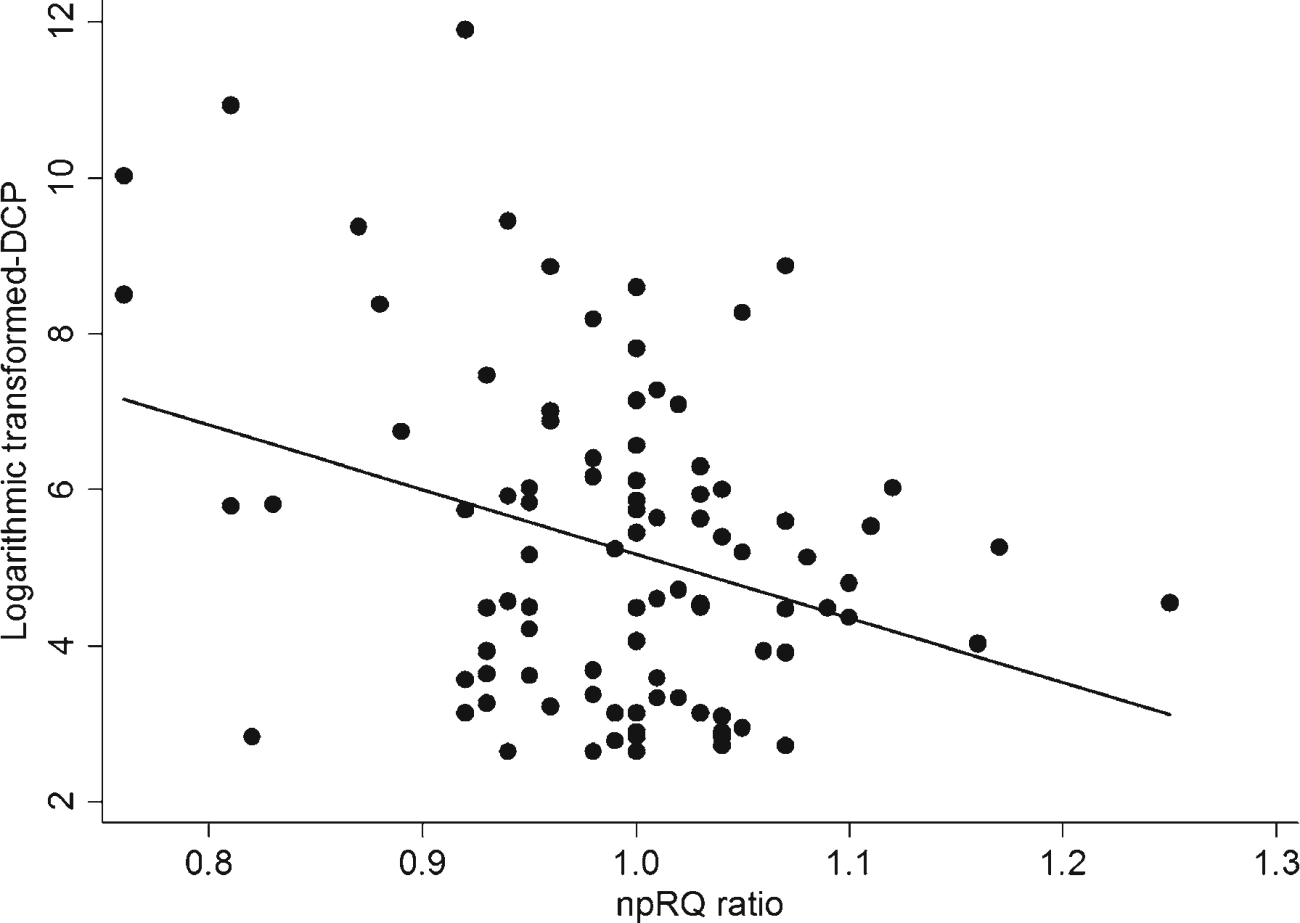
**The correlation between non-protein respiratory quotient (npRQ) ratio (Day 7/Day 0) and des-γ-carboxy prothrombin (DCP) (Day 0) in hepatocellular carcinoma (HCC) patients undergoing transcatheter arterial chemoembolization (TACE).** The npRQ ratio (Day 7/Day 0) was significantly related to des-γ-carboxy prothrombin (DCP) (Day 0) in HCC patients undergoing TACE by Pearson’s correlation coefficient test (p=0.0481, r=-0.2033).

Spearman’s correlation coefficient test showed that npRQ ratio (Day 7/Day 0) was significantly related to des-γ-carboxy prothrombin (DCP) (Day 0) (Figure 1: p=0.0481, r=-0.2033). The npRQ ratio was not related to any other blood biochemical factors (Day 0) (platelets, lymphocytes, aspartate aminotransferase, alanine aminotransferase, γ-glutamyltranspeptidase, total bilirubin, cholinesterase, albumin, prealbumin, branched-chain amino acid/tyrosine ratio, C-reactive protein, glucose, insulin, HOMA-IR, hemoglobin A1c, indocyanine green test retention rate at 15 min, type 4 collagen 7S, hyaluronic acid, and alpha fetoprotein) or the therapeutic volume (p>0.05). In addition, the npRQ ratio was not also related to alpha fetoprotein (Day 0) (p=0.6254, r=-0.0494).

In general, DCP is an HCC tumor marker (Liebman et al. 1984). In this study, DCP was significantly related to the TNM stage and BCLC stage of HCC (p=0.0072 and 0.0001, respectively). On the other hand, DCP production is stimulated by hypoxia in human samples (Murata et al. 2010). Liver cirrhosis is associated with a state of hypoxia (Le Couteur et al. 1999). In liver cirrhosis, the increased resistance to blood flow and oxygen delivery due to an organized basement membrane results in hypoxia (Le Couteur et al. 1999). A recent study showed that serum DCP level was raised in cirrhotic patients without HCC, especially among liver transplant candidates (Yamashiki et al. 2011). Our previous study also showed that HCC patients against a background of liver cirrhosis with a high level of DCP before TACE would become sensitive to hypoxia, and that the tolerability for hypoxia of non-tumoral liver after TACE would decrease (Saito et al. 2012b). In addition, a previous study showed that the severe hypoxic state in the liver parenchyma would influence the decrease of npRQ (Mimura & Furuya 1995). In this study, npRQ reduction after TACE was not affected by the treated size of HCC (p>0.05). It was suggested that cancer cells did not play any positively supportive roles of energy metabolism. Therefore, it was suggested that the HCC patients against a background of cirrhosis with a severe hypoxic state would easily develop npRQ reduction after TACE.

In conclusion, npRQ reduction after TACE was related to a high value of DCP before TACE. The energy malnutrition after TACE would originate from low tolerability for hypoxia of non-tumoral liver. The HCC patients with a high value of DCP before TACE would clinically have a high risk of the energy malnutrition after TACE.

## Abbreviations

AFP: Alpha fetoprotein
DCP: Des-γ-carboxy prothrombin
HCC: Hepatocellular carcinoma
npRQ: Non-protein respiratory quotient
TACE: Transcatheter arterial chemoembolization.
